# County Hospital Administration at Worcester

**Published:** 1913-08-16

**Authors:** 


### The Hospital

The Workers' Newspaper of Administrative Medicine and Institutional Life, Administration, National Insurance and Health.

No. 1416, Vol. LIV. SATURDAY, AUGUST 16, 1913. PRICE ONE PENNY

### COUNTY HOSPITAL ADMINISTRATION AT WORCESTER.

The latest, and on the whole probably the worst, instance of grave incapacity in the financial administration of a hospital is that of the Worcester General Infirmary. In The Hospital of April 16, 1910, page 89, we published a report on the condition of this institution, in which attention was called to the faulty finance and lack of energy in the administration as exhibited by the fact that in twenty-five years, mainly owing to an absence of business ability and energy on the part of the committee, £21,463 had been taken from the invested funds to make good the deficiencies in income. The fact of these annual deficiencies condemns the management and calls for a radical change in the *personnel* of the committee. The hospital generally was in good order, owing to the excellent service rendered by the resident staff, who had to carry on their work under discouraging conditions. These conditions should not exist in any voluntary hospital where the management is energetic and capable. Our inspector pointed out that "the House and Finance Committees had the remedy in their own hands, for the amount of invested funds in 1910 was sufficient to warrant them in putting the whole institution into thorough order, if they so decided. The next step for them to take was to reconsider the whole financial scheme of the hospital and to reform it. The scale of privileges for governors was ridiculously high, seeing that an in-patient ticket is issued for every guinea subscription, or, in other words, every person who subscribes a guinea to the hospital secures the right of taking £5 in value from its funds. This is neither philanthropy nor business, and the position was made infinitely worse from the fact that the invested funds were being slowly but surely dissipated for want of business reform and business management."

These criticisms were not taken in good part, and no effective steps to stay the financial ruin of the Worcester Infirmary were attempted, although the precedent and results of the Shrewsbury, Addenbrooke's and Bath Committee's action, and the success which attended it, should have aroused the Worcester authorities to repentance, and have stayed the rake's progress of the Worcester Committee. Instead of this things were allowed to drift; nothing really practical was attempted, and by the end of 1912, in consequence of the somnolence of the committee, the Worcester Hospital had encroached still further upon its invested funds to the extent of several thousands of pounds. Then the monthly committee declared a financial crisis had arisen and appointed a sub-committee to deal with it. This committee's report is an elaborate condemnation of the whole policy, or want of policy, of the Worcester Infirmary authorities. It contained no suggestion, practical or otherwise, for increasing the revenue, but proposed that the medical, nursing, and domestic staff should be decreased in number, that the salaries of the chaplain, matron, secretary, and dispenser should be reduced, and that in this way £2,000 a year in savings should be effected. The sub-committee further pointed out that if these damaging retrenchments were approved there would still be a drain of some £2,000 a year on the invested funds. We are glad to note that this impotent report aroused the just criticism of Alderman Leicester, Canon Longhurst, Mr. Sydney Harris, Mr. Boyce, and the Hon. Mrs. Britten, to their infinite credit, but impotence triumphed by seventeen votes to seven. The fact that only twenty-four governors attended this important meeting, when the local papers record that over fifty persons attended a meeting of the County Cricket Club, seems to indicate that the inhabitants of Worcester are not unnaturally disgusted and utterly out of sympathy with the present committee of the infirmary.

The financial position of the Worcester Infirmary could be made perfectly sound in the course of six months by the appointment of a new committee of men of business and women of enterprise, like the Hon. Mrs. Britten. Instead of reducing the secretary's salary by 20 per cent., the services of a trained and competent hospital administrator as

Secretary-Superintendent, at an adequate salary, should be secured, who will devote his whole time and energies to the work of the hospital. The present regulations defining the privileges of the governors should be entirely remodelled, and the scale of privileges largely reduced. A system of paying wards should be established to meet the cases suggested by Canon Longhurst and to enable the whole of the beds in the hospital to be brought into active use, and so be made of service to the whole community. These changes would, in our judgment, speedily place the Worcester General Infirmary upon a sound financial basis, secure for it the widespread interest of the community it was originally established to serve, re-establish public confidence in the institution, and prevent the damaging effect upon the nurse-training school and the resident medical staff which the past policy of the present committee, unless it be at once reversed, must inevitably produce. It is not possible to give an adequate training or to conduct a modern training school for nurses upon an efficient basis in an institution where the number of beds maintained has been allowed to fall as they have done at Worcester. It is already difficult enough to secure competent candidates for the post of resident medical officer in provincial hospitals, but the condition of affairs at the Worcester Infirmary, as revealed by the recent reports of the meetings and proceedings of the governors and committees to which we have referred, cannot fail to prevent the authorities of medical schools from recommending their students to offer themselves for posts on the resident medical staff.

We hope, therefore, that those who spoke and voted against the feeble policy recommended by the sub-committee will meet and proceed to form a large committee of influential city and county residents, with the object of bringing pressure to bear upon the existing members of the committee, and to secure the retirement of the majority who may be responsible for the present failure to administer the affairs of the Worcester Infirmary with business acumen and vigorous energy. We say this without knowing the name of a single member of the present committee, and we recommend this course on public grounds, basing our judgment on the evidence the committee has so forcibly produced in support of its own condemnation by exhibiting probably the greatest failure ever secured by a hospital committee in this country.

